# Supplementary material for: Direct observation of single-molecule hydrogen-bond dynamics with single-bond resolution
Source: Nat Commun. 2018 Feb 23;9:807. doi: 10.1038/s41467-018-03203-1 (PMC5825177; doi:10.1038/s41467-018-03203-1)
Supplement: Supplementary file 1 — Supplementary Information [file 41467_2018_3203_MOESM1_ESM.pdf]

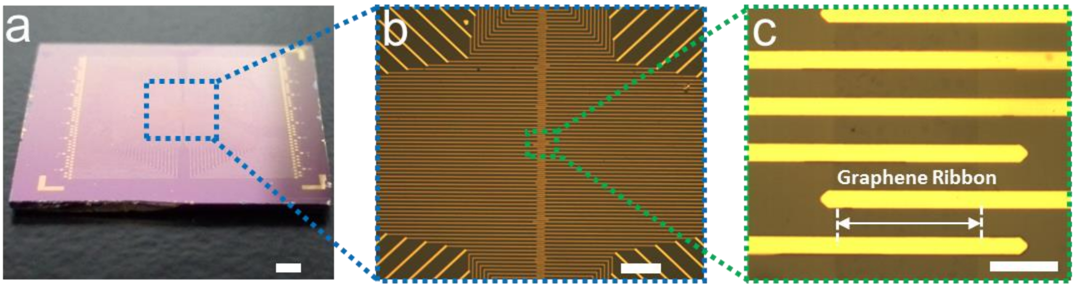

Supplementary Figure 1 | Optical images of graphene devices with different magnifications. The devices with graphene point contact arrays were fabricated by a dash-line lithography (DLL) method as described in a previous publication[1]. The yellow part is deposited gold electrodes (Cr/Au: 8/60 nm), and the centre part under gold electrodes is the graphene ribbon (40 × 150 μm^2). Scale bar: a, 1 mm; b, 100 μm; c, 20 μm. After the DLL process, the freshly prepared devices with a graphene point contact array were immersed in a pyridine solution containing 0.1 mM of Compound 1 for coupling reactions in the presence of 1 mM EDCI. After reaction for 48 h, the devices were removed from the solution and rinsed with deionised H_2O and Me_2CO several times before being dried with a N_2 stream.

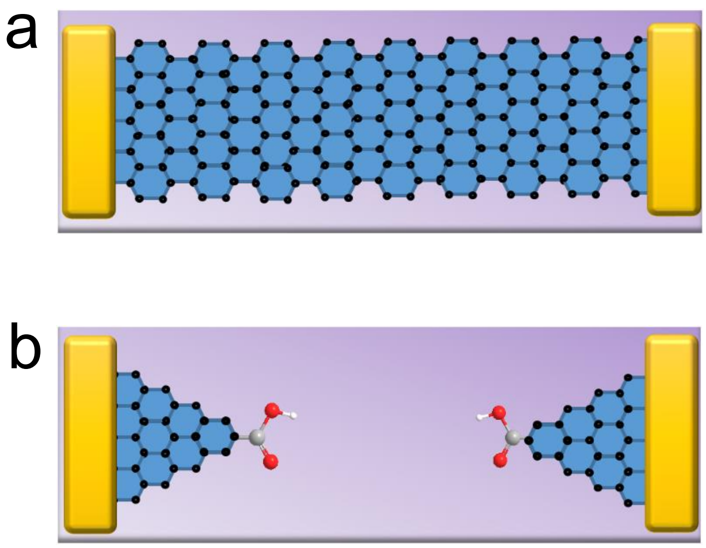

Supplementary Figure 2 | Schematic of fabricating molecular devices. a, Schematic representation of a pristine graphene device transferred from the PMMA support. **b**, A device with a pair of graphene point contacts prepared by using the DLL process and oxygen plasma etching.

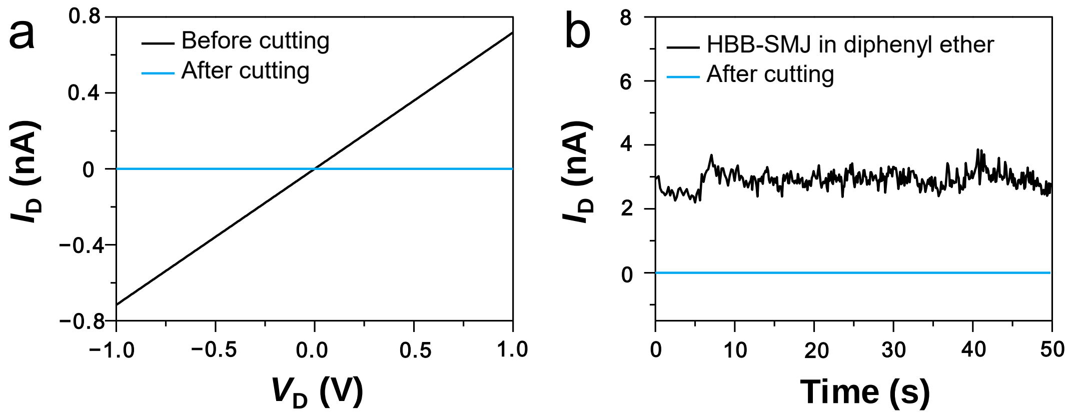

Supplementary Figure 3 | Device characteristics of HBB-SMJs at the different stages. a, *I*-*V* curves of a graphene device before (black) and after (blue) e-beam lithographic cutting. **b**, *I*-*t* curves of a representative graphene device (V_{bias} = 50 mV) after e-beam lithographic cutting (blue) and molecular connection (black), respectively. All data were collected using the Agilent characterisation system. Preliminary electrical characterisation (*I*-*V* and *I*-*t*) was carried out at room temperature in the ambient atmosphere by using an Agilent 4155C semiconductor parameter system (DC measurements) and a Karl Suss (PM5) manual probe station.

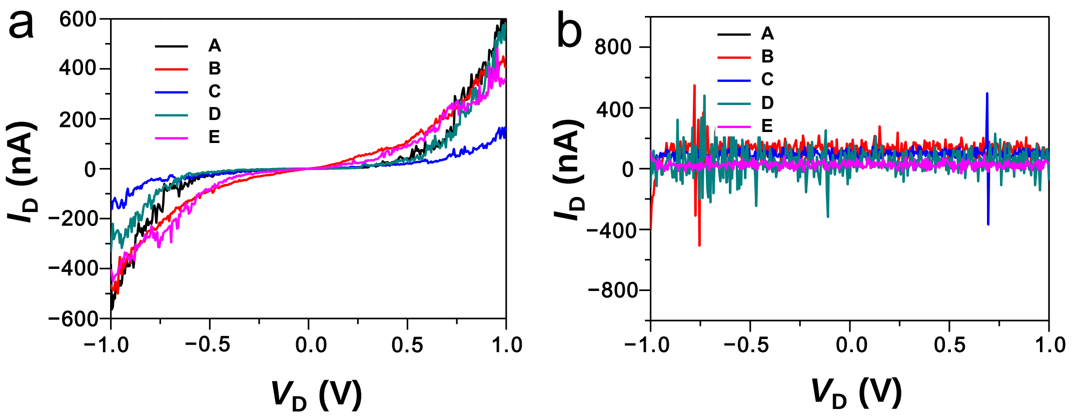

Supplementary Figure 4 | Reproducibility. *I*-*V* curves measured by using the Agilent characterisation system on another five devices in diphenyl ether (**a**) and in water (**b**).

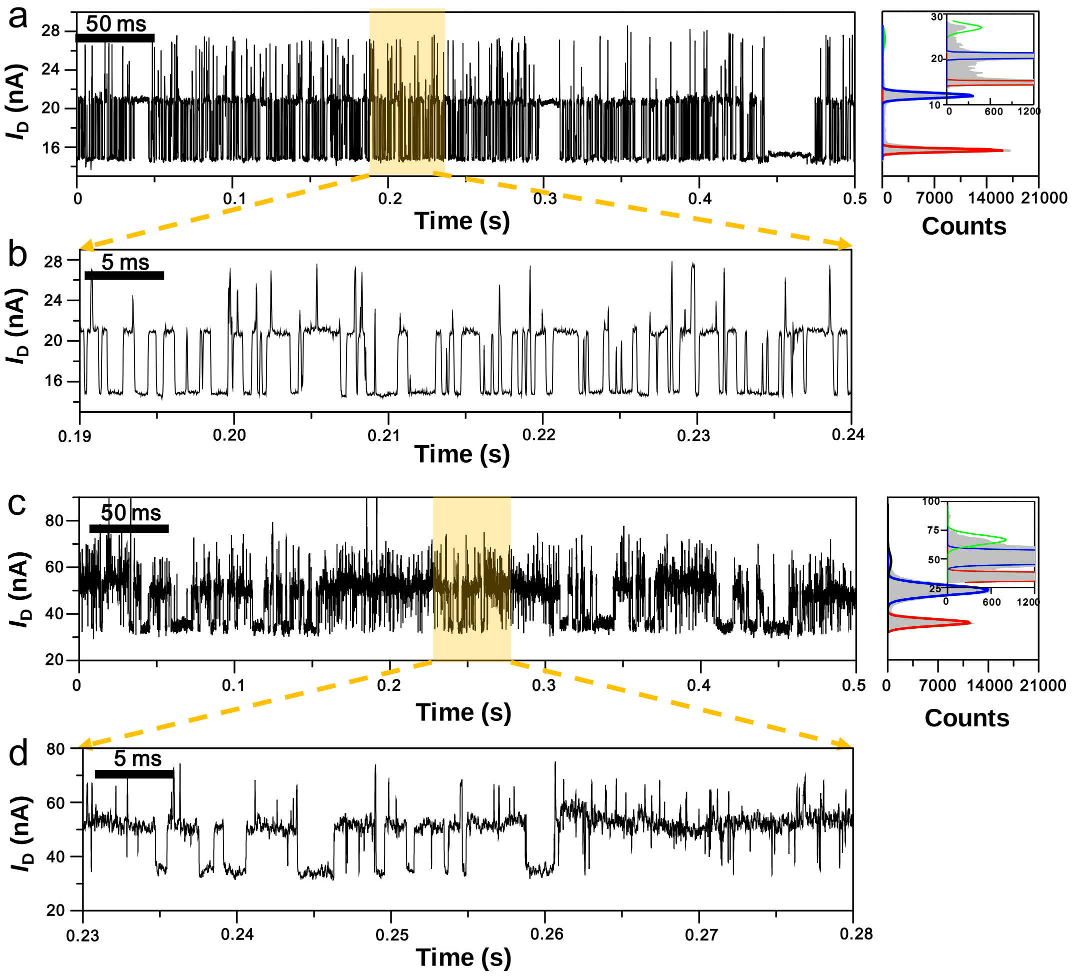

Supplementary Figure 5 | Real-time recordings of HBB-SMJs at 323 K in diphenyl ether. a-b, another set of data without State 2 in the same device in Fig. 3. **c-d**, Additional set of data at 323 K in diphenyl ether. V_{bias} = 300 mV.

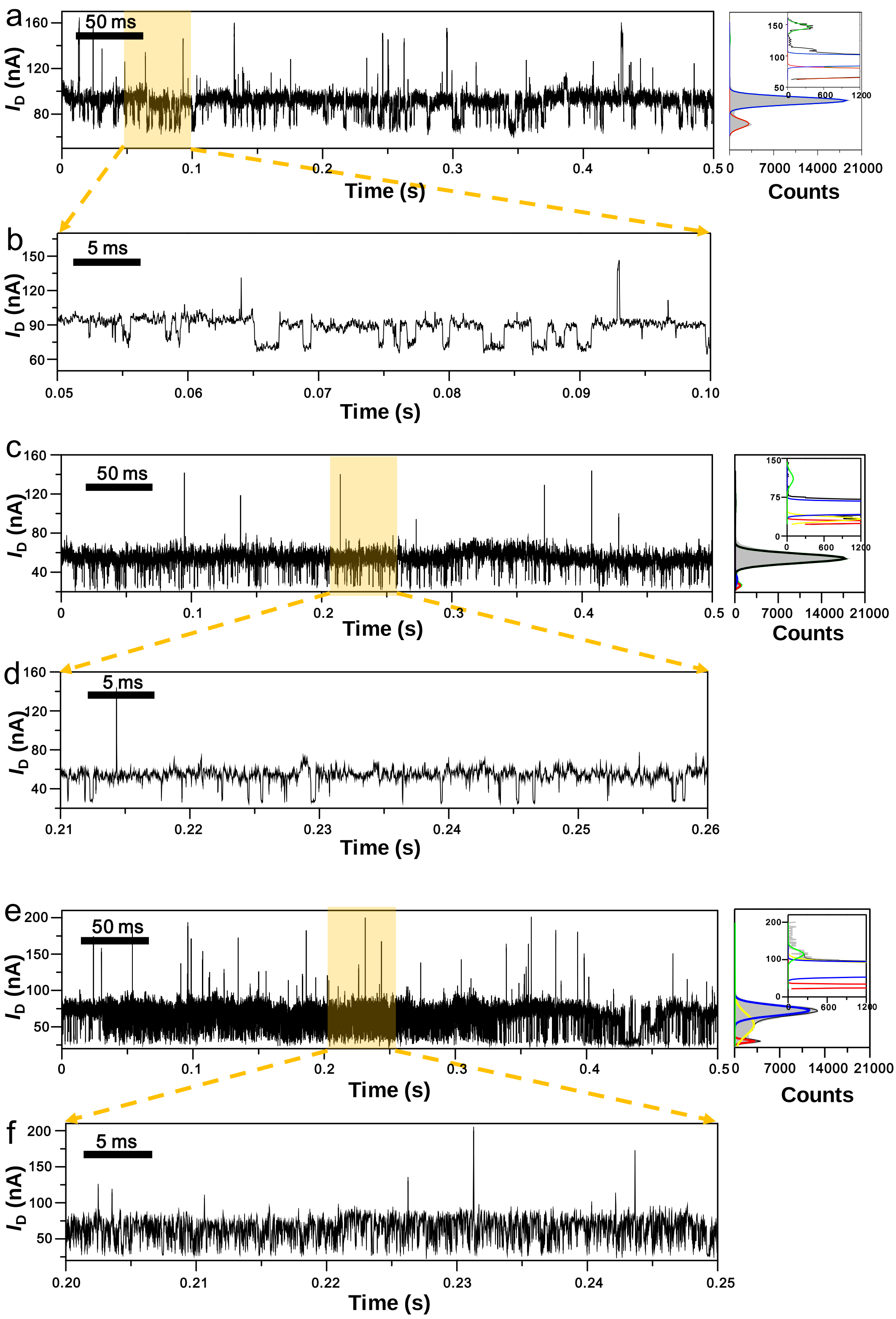

Supplementary Figure 6 | Real-time measurements of hydrogen-bond dynamics in HBB-SMJs in diphenyl ether at 323 K (a-b), 333 K (c-d), 343 K (e-f). V_{bias} = 300 mV.

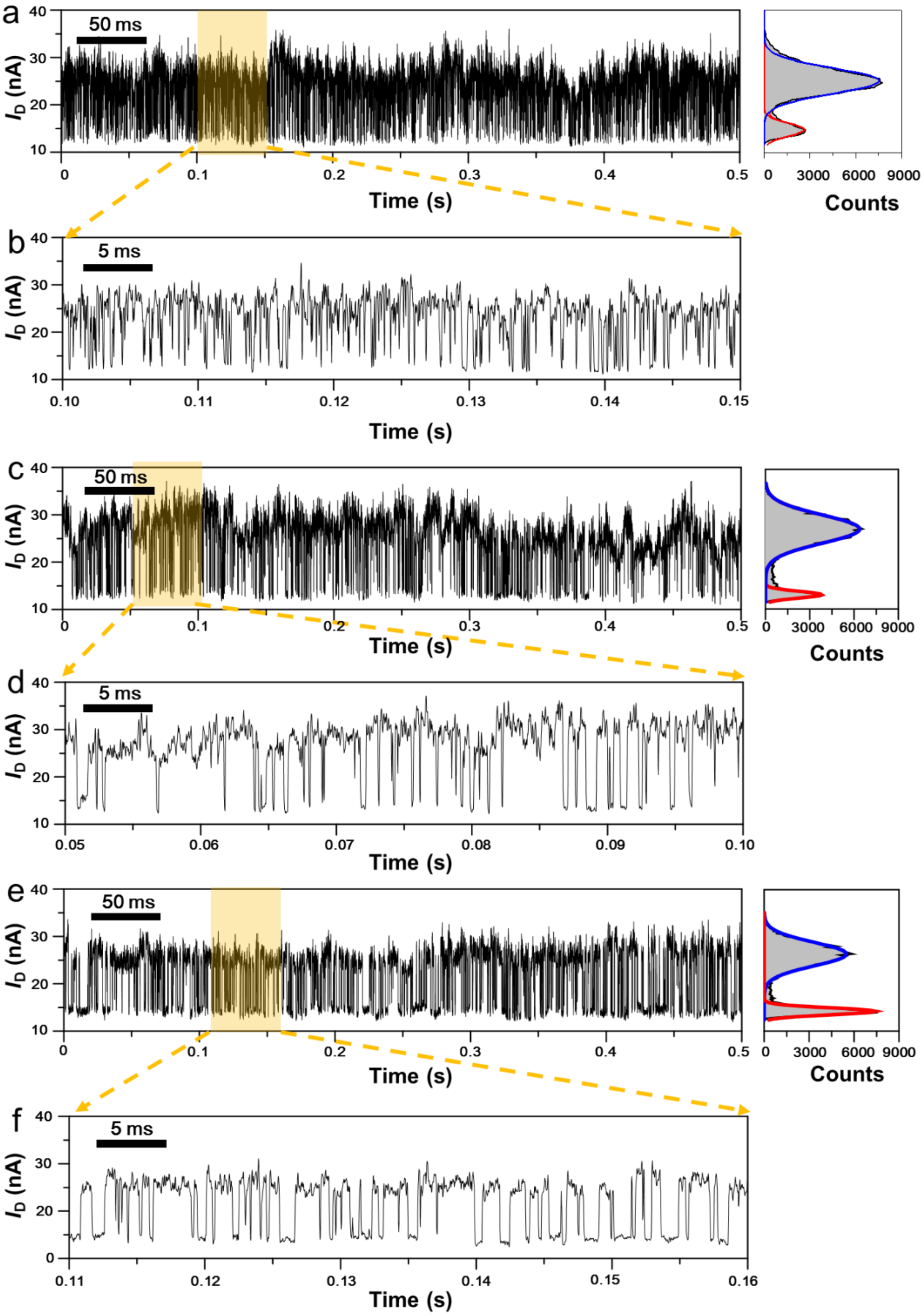

Supplementary Figure 7 | Real-time measurements of hydrogen-bond dynamics in HBB-SMJs in TeCA at 273 K (a-b), 293 K (c-d), 313 K (e-f). V_{bias} = 300 mV.

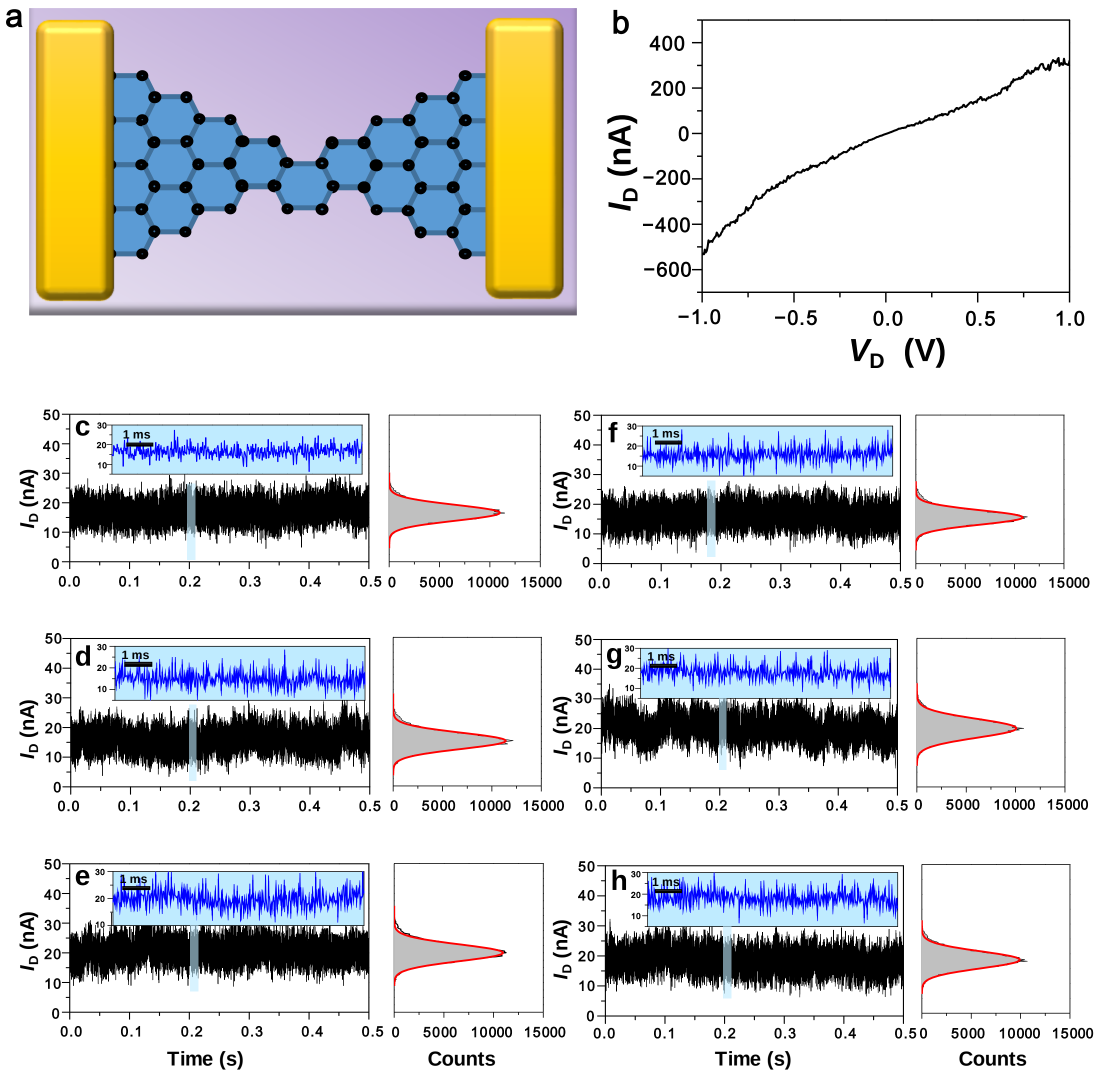

Supplementary Figure 8 | Control experiments by using a partially-cleaved graphene ribbon device. a, Schematic representation of the partially-cleaved graphene ribbon device. **b**, *I-V* curve of the control device in diphenyl ether. **c-e**, *I-t* curves of the control device immersed in TeCA at 273 K (**c**), 293 K (**d**) and 313 K (**e**). **f-h**, *I-t* curves of the control device immersed diphenyl ether at 323 K (**f**), 333 K (**g**) and 343 K (**h**). V_{bias}= 300 mV.

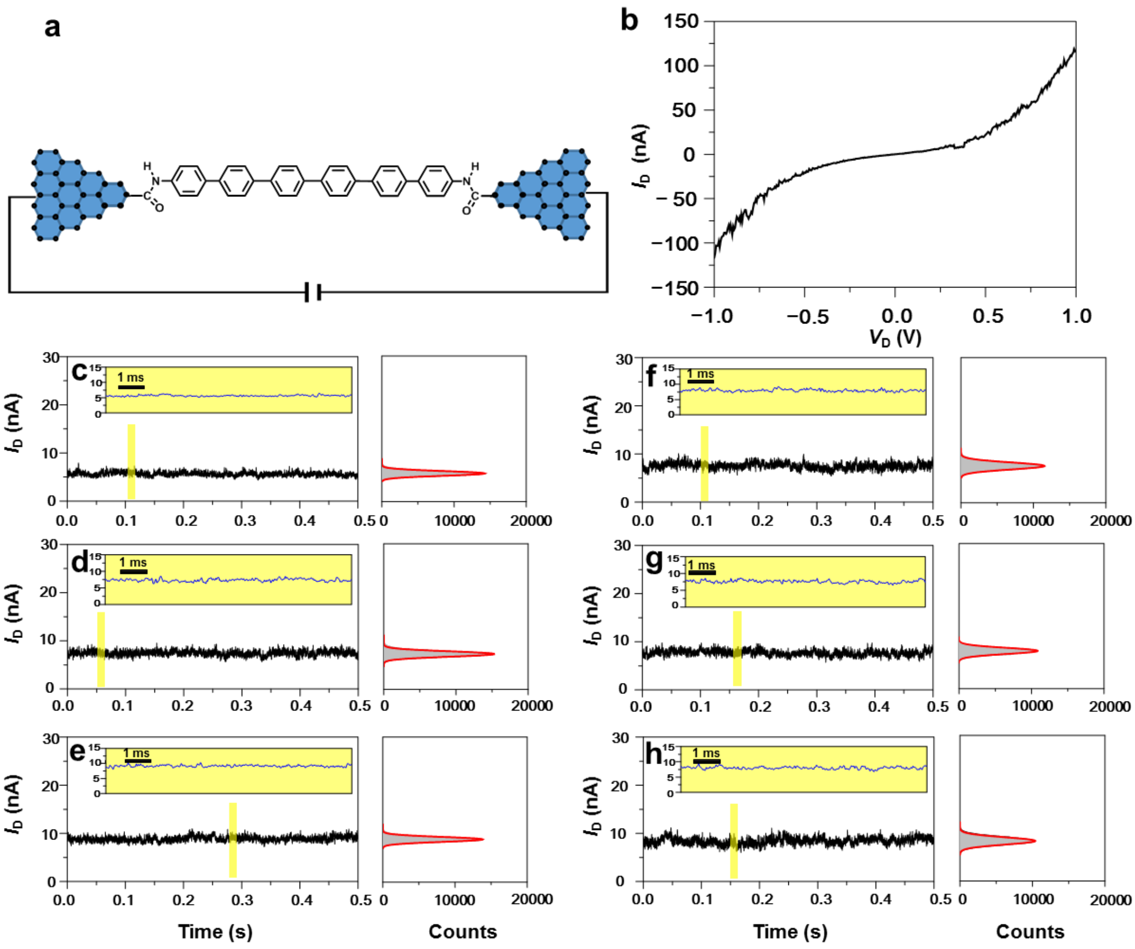

Supplementary Figure 9 | Control experiments by using a control molecule: [1,1’:4’,1”:4”,1’”:4’”,1””:4””,1’””-Sexiphenyl]-4,4’””-diamine. **a**, Schematic representation of a control device. **b**, *I*-*V* curve of the control device in diphenyl ether. **c-e**, *I*-*t* curves of the control device immersed in TeCA at 273 K (**c**), 293 K (**d**) and 313 K (**e**). **f-h**, *I*-*t* curves of the control device immersed in diphenyl ether at 323 K (**f**), 333 K (**g**) and 343 K (**h**). V_{bias}= 300 mV.

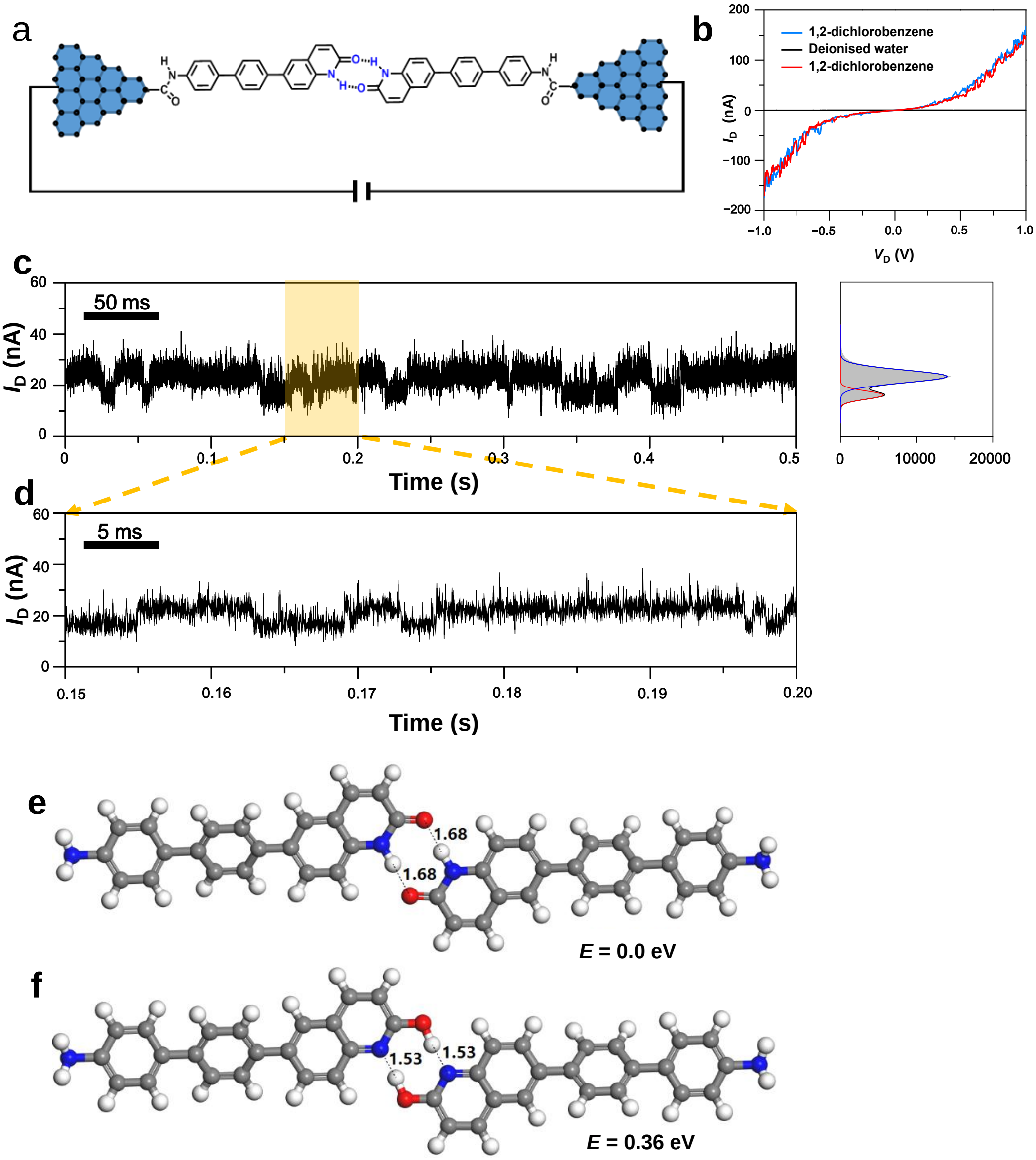

Supplementary Figure 10 | Device structure and electrical characterisation of HBB-SMJs with a double hydrogen-bond system. a, Schematic representation of the HBB-SMJ device by using 6-(4'-amino-[1,1'-biphenyl]-4-yl)quinolin-2(1H)-one. **b**, *I*-*V* curves of a HBB-SMJ sequentially immersed in 1,2-dichlorobenzene (blue), deionised water (black) and 1,2-dichlorobenzene again (red). **c-d,** Real-time measurements of hydrogen-bond dynamics in HBB-SMJs in 1,2-dichlorobenzene at 293 K. V_{bias} = 300 mV. **e-f,** The intermolecular proton transfer model. The calculation results indicated that the high-condutance state belongs to the stucture in **(e)** with the low energy and long dwell times and the low-conductance state belongs to the structure in **(f)** with the high energy and short dwell times.

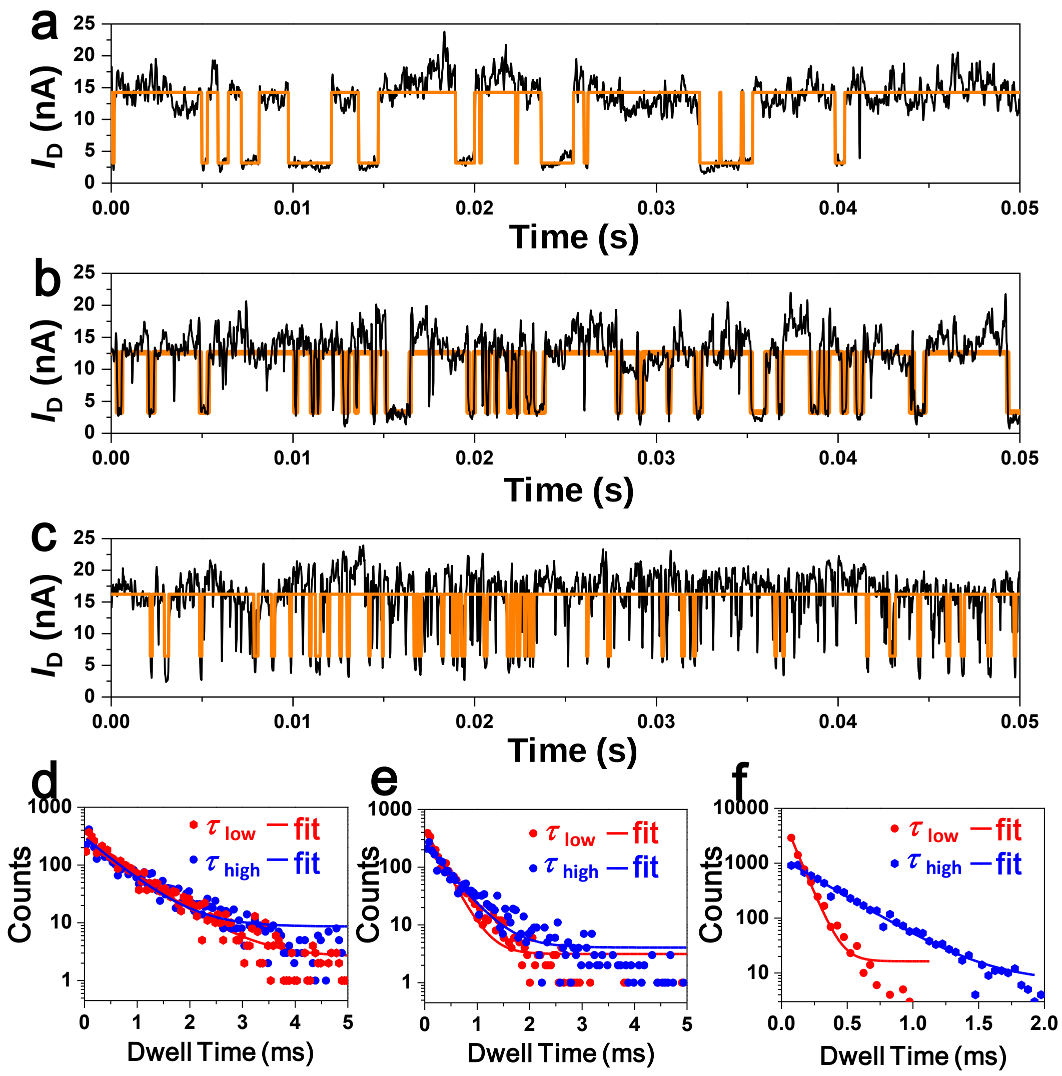

Supplementary Figure 11 │ kinetics analyses in TeCA. Full raw *I-t* data in TeCA were idealised according to a Hidden-Markov model (HMM) by using a QUB software. This idealised data were further analysed to provide one set of kinetic parameters of the HBB-SMJ devices, including the lifetimes τ_{high} and τ_{low}. **a-c**, *I-t* curves (black) of a HBB-SMJ device in TeCA at different temperatures (273 K, 293 K, 313 K, from top to bottom), and the idealised fitting (orange) obtained from a segmental *k*-means (SKM) method based on a Hidden-Markov-model analysis by using a QUB software. V_{bias} = 300 mV. **d-f**, Plots of time intervals of the "high" (τ_{high}, blue) and "low" (τ_{low}, red) current states in the idealised fitting in (**a-c**), and the corresponding exponential fittings in which their lifetimes (τ_{high} and τ_{low}) can be derived.

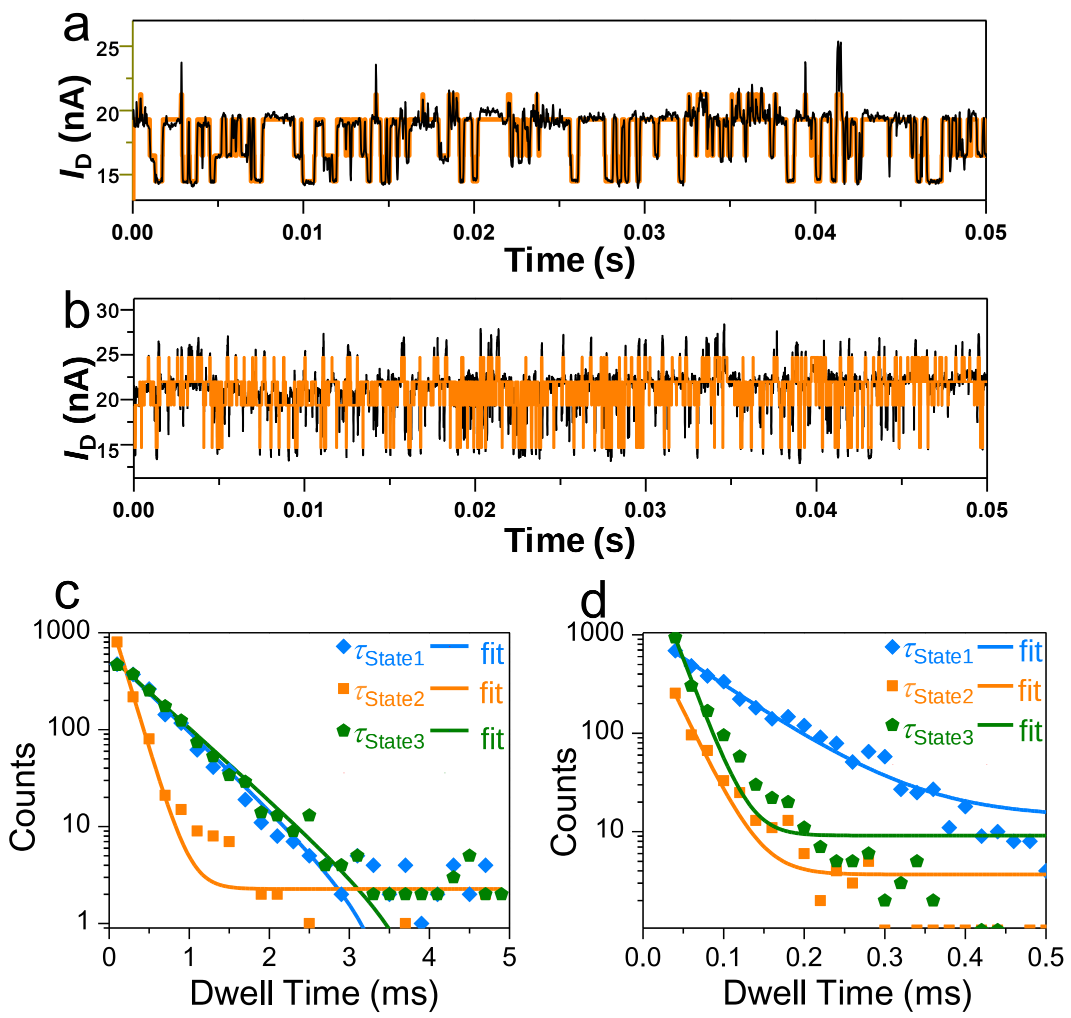

Supplementary Figure 12 | kinetics analyses in diphenyl ether. a-b, *I-t* curves (black) of a HBB-SMJ device in diphenyl ether at different temperatures (323 K and 333 K, from top to bottom), and the idealised fitting (orange) obtained from a segmental *k*-means (SKM) method based on a Hidden-Markov-model analysis by using a QUB software. V_{bias} = 300 mV. **c-d**, Plots of time intervals of States 1−3 in the idealised fitting in (**c**) and (**d**), and the corresponding exponential fittings in which their lifetimes ($\tau_{State\ 1}$, $\tau_{State\ 2}$ and $\tau_{State\ 3}$) can be derived.

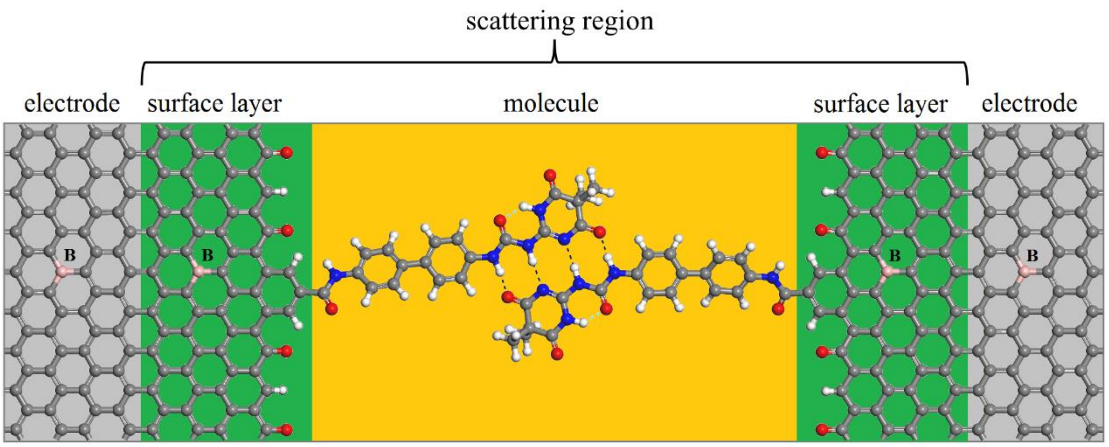

Supplementary Figure 13 ｜ The transport device model. To save computational cost, the butyl groups in the central molecule are reduced to methyl groups.

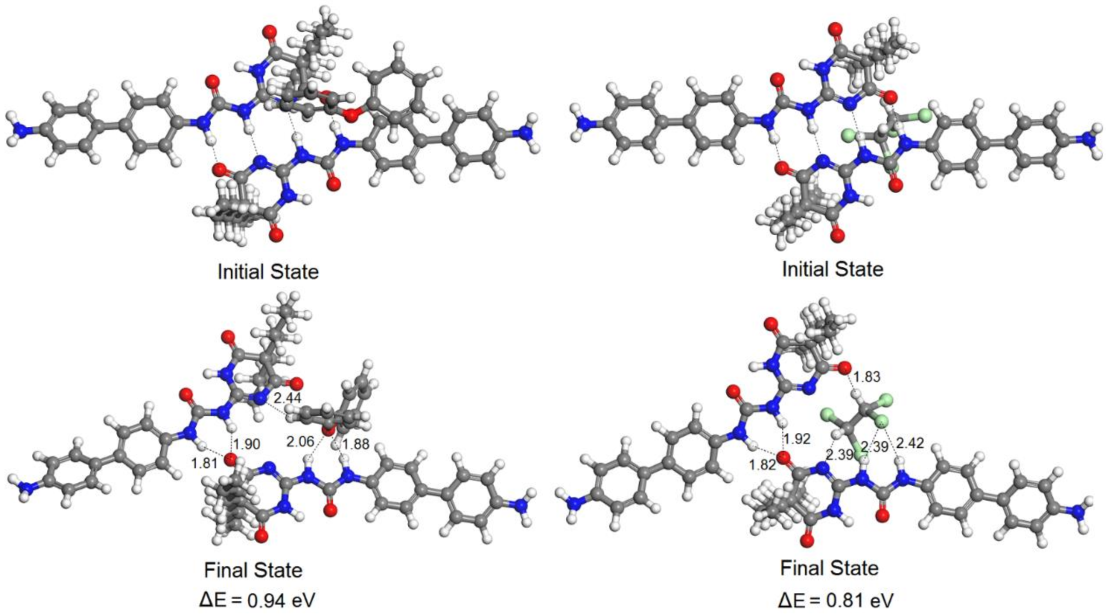

Supplementary Figure 14 ｜ The intercalation model.

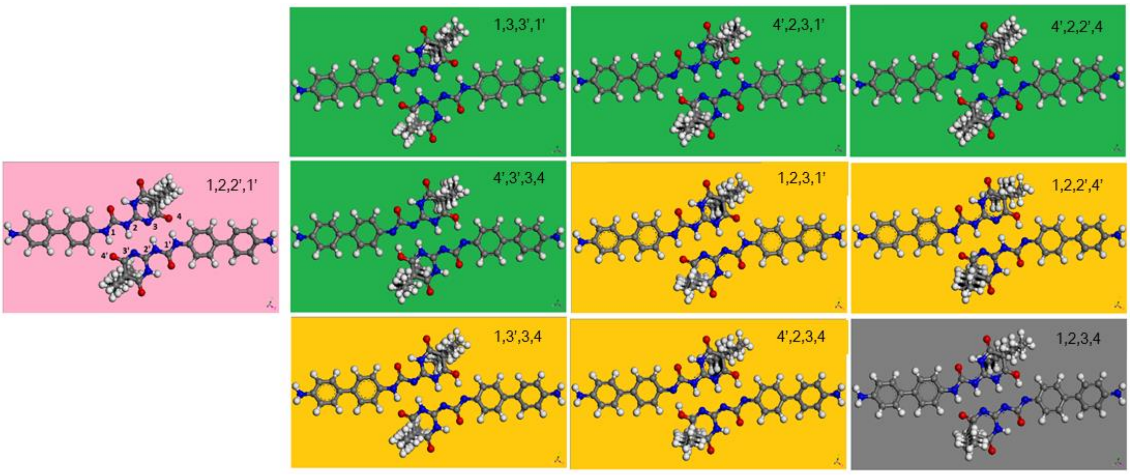

Supplementary Figure 15 | The possible configurations induced by intermolecular proton transfer reactions. The pink one (1,2,2',1') is the most stable state. The next stable state is 1,3,3',1', and the third stable state is 1,2,3,1', which are located just 0.06 and 0.35 eV above the 1,2,2',1 basic state, respectively. Other configurations are found at least 1.0 eV higher in energy than the 1,2,2',1' basic state.

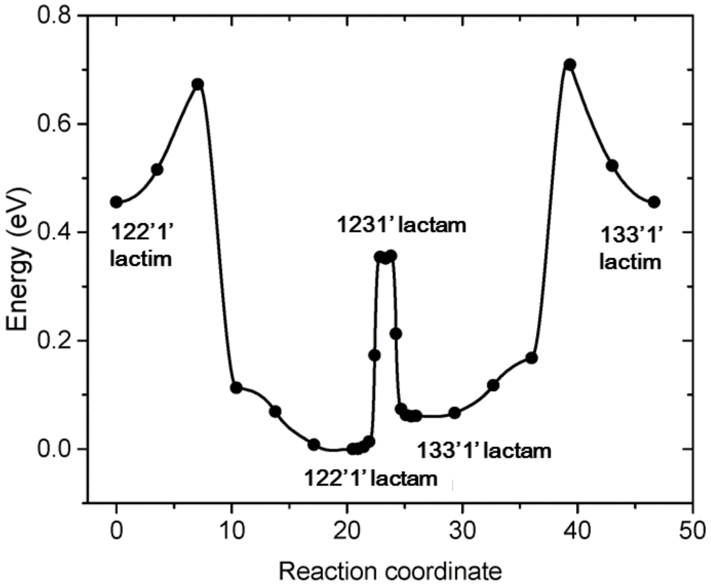

Supplementary Figure 16 | The lowest energy reaction pathway of hydrogen-bond transformations in diphenyl ether at 323 K. Within the NEB calculations, the reaction coordinate is defined as the distance of each image structure to the 122'1' lactim structure on the potential energy surface. Depending on the strength of hydrogen bond and its chemical environment, previous estimations of the barriers for intermolecular proton transfer vary from 1 kcal/mol to about 20 kcal/mol, i.e. 0.04 eV to 0.86 eV[13–15], while for lactam-lactim tautomerism, the energy barriers could change from 7 kcal/mol to 40 kcal/mol, i.e. 0.30 eV to 1.73 eV[16,17]. In our work, the calculated barriers for intermolecular proton transfer (0.35 eV) and lactam-lactim tautomerism (0.67 eV) fall reasonably in the range of previous estimations.

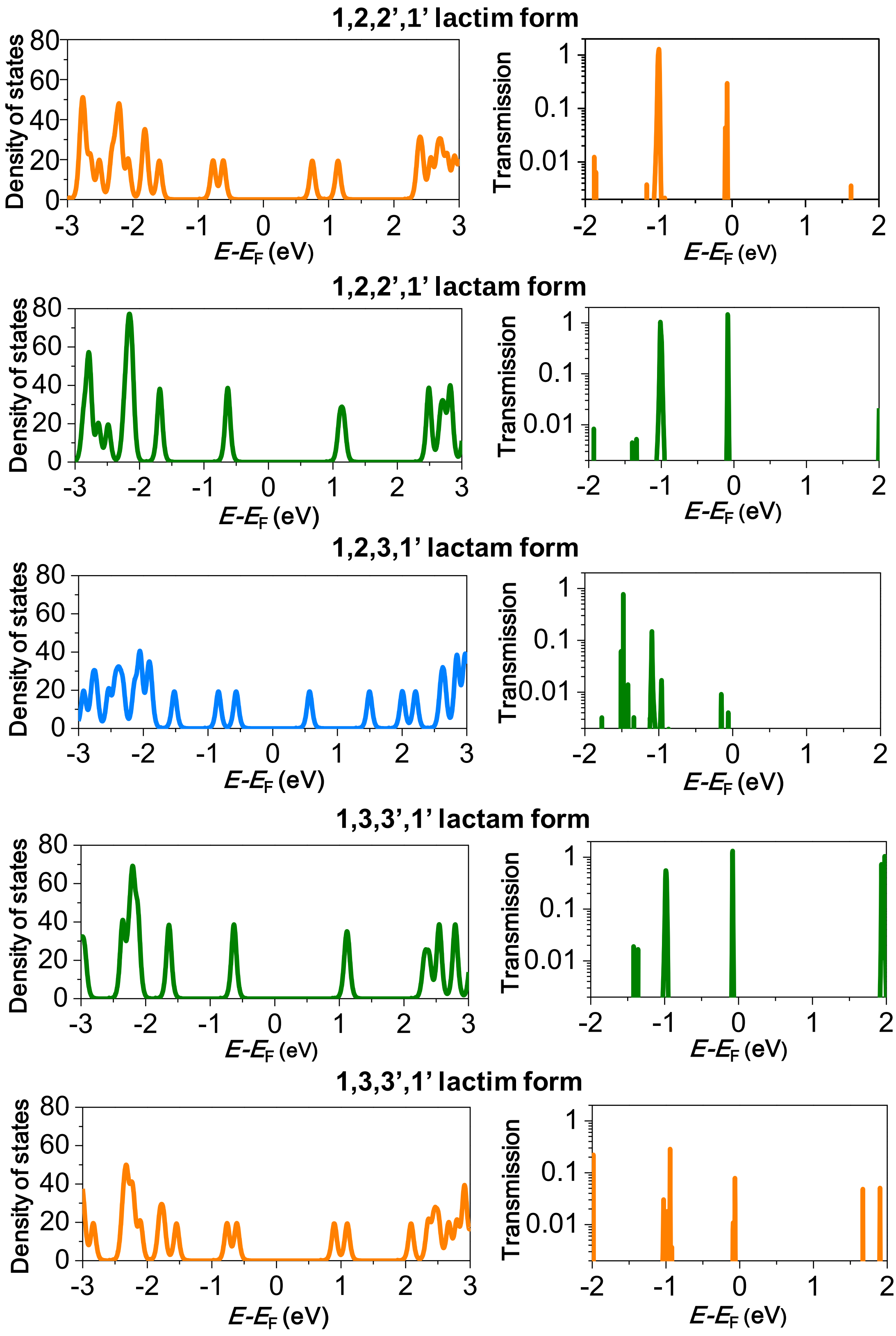

Supplementary Figure 17 | The density of states and transmission spectra of five low-lying energy microstates.

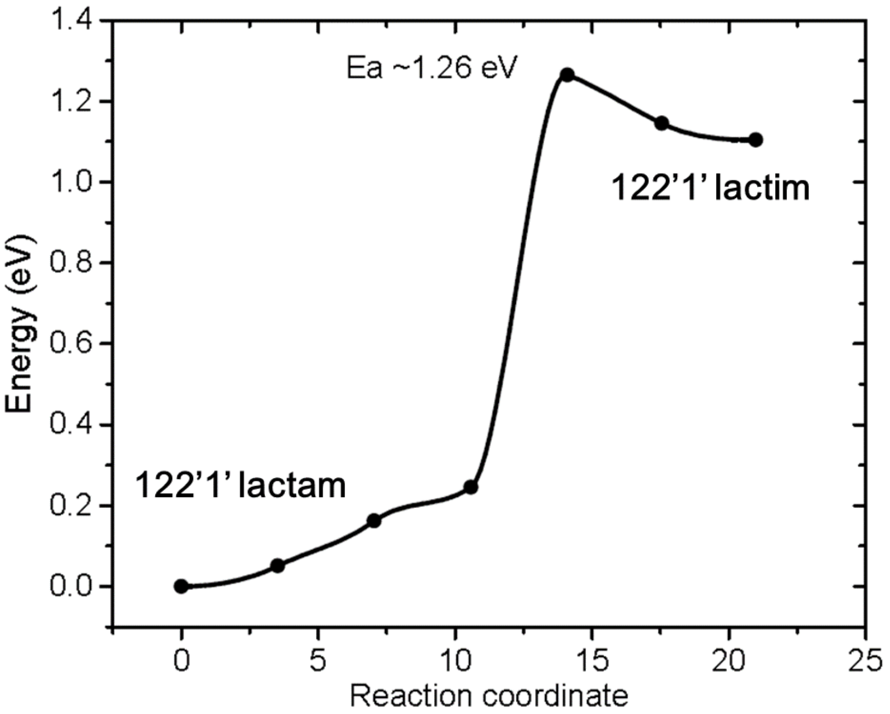

Supplementary Figure 18 | The lowest energy reaction pathways of the lactam-lactim tautomerism process in TeCA at 313 K. Within the NEB calculations, the reaction coordinate is defined as the distance of each image structure to the 122'1' lactam structure on the potential energy surface.

Supplementary Table 1: The lifetimes (τ_{high} and τ_{low}) and the corresponding interconversion rate constants in TeCA.

T (K)	273	293	313
τ_{low} (ms)	0.581±0.042	0.253±0.018	0.075±0.001
τ_{high} (ms)	0.660±0.042	0.393±0.019	0.300±0.014
k_1 (s^{-1})	1721±124	3953±281	13333±178
k_2 (s^{-1})	1515±96	2545±123	3333±155

Supplementary Table 2: The lifetimes of States 1−3 and the corresponding interconversion rate constants in diphenyl ether.

	T (K)	323	333	343
Total	$\tau_{State\ 1}$ (ms)	0.545±0.022	0.078±0.004	N/A
	$\tau_{State\ 2}$ (ms)	0.194±0.022	0.020±0.001	N/A
	$\tau_{State\ 3}$ (ms)	0.585±0.018	0.026±0.001	N/A
State 3→State 1	$P_{State\ 3\to1}$	64%	56%	N/A
	$\tau_{State\ 3\to1}$	0.514±0.020	0.021±0.001	N/A
	$k_{State\ 3\to1}$ (ms^{-1})	1.945±0.075	47.62±2.267	N/A
State 3→State 2	$P_{State\ 3\to2}$	36%	44%	N/A
	$\tau_{State\ 3\to2}$ (ms)	0.752±0.048	0.036±0.001	N/A
	$k_{State\ 3\to2}$ (ms^{-1})	1.330±0.084	27.78±0.771	N/A
State 2→State 3	$P_{State\ 2\to3}$	96.2%	99.6%	N/A
	$\tau_{State\ 2\to3}$ (ms)	0.186±0.018	0.020±0.001	N/A
	$k_{State\ 2\to3}$ (ms^{-1})	5.376±0.52	50±2.500	N/A
State 1→State 3	$P_{State\ 1\to3}$	96.5%	99.3%	N/A
	$\tau_{State\ 1\to3}$ (ms)	0.561±0.029	0.079±0.002	N/A
	$k_{State\ 1\to3}$ (ms^{-1})	1.783±0.092	12.66±0.320	N/A
Rare Event	$P_{State\ 1\to2}$	3.5%	0.7%	N/A
	$P_{State\ 2\to1}$	3.8%	0.4%	N/A

P is the possibility of the interconversion occurrence for each state. It is difficult to extract the lifetimes and corresponding interconversion rate constants for the interconversion between State 1 and State 2 because these events are very rare.

Supplementary Note 1. Synthetic Methods

NMR spectra were recorded in the designated solvent on Bruker Avance 400 MHz spectrometer. Spectra were reported in ppm values from residual protons of deuterated solvent. Mass data were obtained with a Bruker Daltonics Inc. Microanalysis was carried out using Flash EA 1112 or Carlo Erba 1106 analyser at the Institute of Chemistry, Chinese Academy of Sciences.

Synthesis of 1-(4'-amino-[1,1'-biphenyl]-4-yl)-3-(5,5-dibutyl-4,6-dioxo-1,4,5,6-tetrahydropyrimidin-2-yl)urea

The crude materials of 4'-nitro-[1,1'-biphenyl]-4-amine were purchased from commercial corporation. 2-amino-5,5-dibutylpyrimidine-4,6(1*H*,5*H*)-dione (**3**) was prepared according to the known procedure[2].

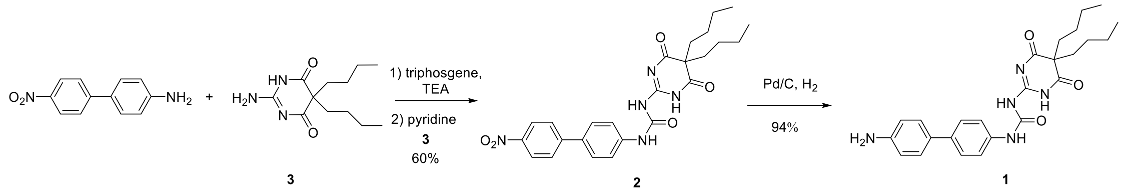

Compound 2: The mixture of 4'-nitro-[1,1'-biphenyl]-4-amine (107 mg, 0.5 mmol), triphosgene (150 mg, 0.5 mmol) in 10 mL dichloromethane was cooled to 0-5°C in the ice bath, and the triethylamine (0.4 mL) was added dropwise, then the solution was stirred for 1 h at 0-5°C. Monitored by TLC, after completion, compound **3** (120 mg, 0.5 mmol) in 5 mL pyridine was added, then the solution was heated to reflux at 90°C for another 2 h. Then, the solution was cooled to room temperature and the solvent was removed under vacuum. This crude product was purified by column chromatography on silica gel (eluent: dichloromethane/ethyl acetate 50:1, v/v) to give desired product 145 mg as a yellow solid in the yield of 60%. ^{1}H NMR (400 MHz, CD_2Cl_2): δ 0.85 (t, J = 6.8 Hz, 6H), 1.27–1.29 (m, overlapping, 8H), 2.01–2.10 (m, overlapping, 4H), 7.67 (d, J = 8.8 Hz, 2H), 7.76 (d, J = 8.8 Hz, 2H), 7.83 (d, J = 8.8 Hz, 2H), 8.27 (d, J = 8.8 Hz, 2H), 10.46 (s, 1H), 11.26 (s, 1H), 12.66 (s, 1H). ^{13}C NMR (100 MHz, CD_3CN + DMSO-d_6): δ 13.8, 22.7, 27.1, 38.57, 56.8, 120.4, 124.5, 127.5, 128.0, 132.8, 140.7, 146.8, 146.9, 150.0, 161.4, 172.5. HR-MS (MALDI-TOF, m/z): calcd. for $C_{25}H_{29}N_5O_5$: 478.2 $[M-H]^+$; Found: 478.2.

Compound 1: To the mixture of compound **2** (120 mg, 0.25 mmol) in 6 mL CH_3OH was added Pd/C (10 wt％, 12 mg, 1 mol％ Pd). The reaction vessel was purged with N_2, then H_2, and pressurised with H_2 to 55 psi. Then, the mixture was stirred at room temperature for 18 h. After completion, the reaction mixture was filtered through Celite. The filter bed was washed with water and THF. Then, the filtrate was concentrated under vacuum. The crude product was purified by column chromatography on silica gel (eluent: CH_2Cl_2/EtOH 100:1, v/v) to give desired product 105 mg as a yellow solid in the yield of 95%. ^{1}H NMR (400 MHz, $CDCl_3$): δ 0.86 (t, J = 6.8 Hz, 6H), 1.24–1.31 (m, overlapping, 8H), 2.05 (t, J = 7.6 Hz, 4H), 3.73 (s, 2 H), 6.76 (d, J = 8.0 Hz, 2H), 7.41 (d, J = 8.0 Hz, 2H), 7.54 (d, J = 8.0 Hz, 2H), 7.69 (d, J = 8.0 Hz, 2H), 10.38 (s, 1H), 11.27 (s, 1H), 12.73 (s, 1H). ^{13}C NMR (100 MHz, $CDCl_3$): δ 13.8, 22.8, 27.4, 39.6, 58.3, 115.6, 120.6, 126.9, 127.9, 131.0, 136.2, 137.6, 145.9, 155.6, 156.4, 172.0, 180.9. ESI-MS (m/z): calcd. for $C_{25}H_{31}N_5O_3$: 450.3 $[M+H]^+$; Found: 450.3. ^{1}H NMR spectra (500 MHz) of Compounds **1** in 1,1,2,2-Tetrachloroethane-d_2 at 313 K showed three singlet signals (12.58, 10.68 and 9.86 ppm) in the lower field and no such three singlet signals were observed in ethanol-d_6.

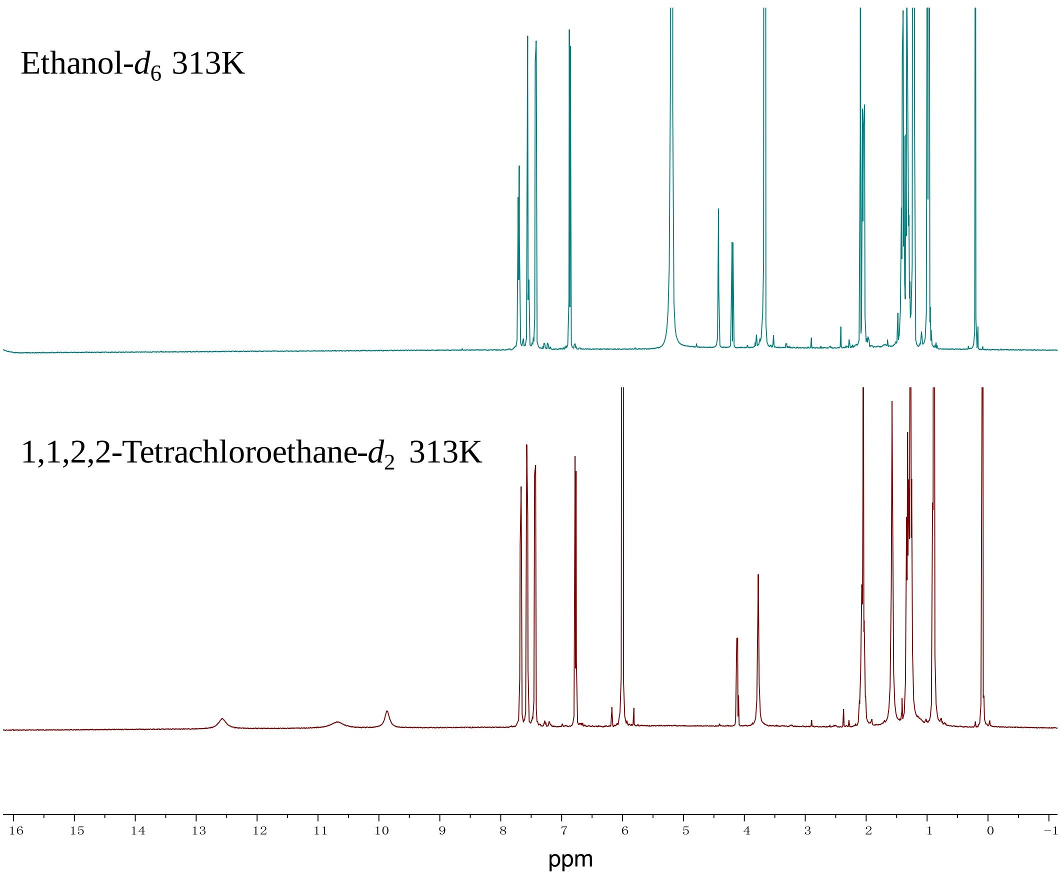

Synthesis of 6-(4'-amino-[1,1'-biphenyl]-4-yl)quinolin-2(1H)-one

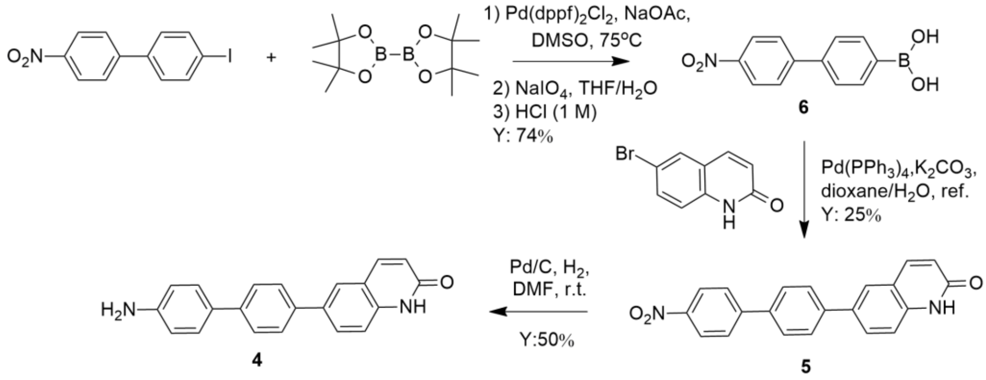

Compound 6: To the mixture of 4-iodo-4'-nitro-1,1'-biphenyl (898 mg, 3 mmol), bis(pinacolato)diboron (1070 mg, 4.2 mmol) and NaOAc (882 mg, 3 mmol) in 16 mL anhydrous DMSO solution was added Pd(dppf)Cl_2 (98 mg, 0.04 mmol) under N_2 atmosphere, then the mixture was bubbled with N_2 for 10 min and heated at 75 ℃ for another 10 h. Monitored by TLC, when complete, the mixture was cooled to room temperature and poured into water (80 mL) and extracted with Et_2O (4 ×50 mL). The

combined organic layers were washed with water (2 × 50 mL), saturated NaCl aqueous solution (50 mL) in sequence, and dried over anhydrous Na_2SO_4, then the solvent was removed by reduced pressure. To the residue were added water (5 mL), THF (5 mL), and $NaIO_4$ (1.93 g, 9 mmol), and the mixture was vigorously stirred at ambient temperature for 1 h, followed by 15 mL aqueous HCl (1 M) was added and stirred additionally for 4 h at ambient temperature. Then extracted with AcOEt (4 ×60 mL). The combined organic layers were washed with water (100 mL) and brine (100 mL), dried over anhydrous $MgSO_4$, and evaporated to dryness. The residue was subjected to column chromatography (eluent: CH_2Cl_2/ethyl acetate 5/1) to give desired pale yellow solid in the yield of 74%. ^{1}H NMR (400 MHz, DMSO-d_6): δ 7.78 (d, J = 7.6 Hz, 2H), 7.99 (dd, J = 8.0, 10.4 Hz, 4H), 8.11 (s, 2H), 8.33 (d, J = 8.0 Hz, 2H). ^{13}C NMR (100 MHz, DMSO-d_6): δ 124.5, 126.7, 128.3, 135.4, 139.6, 147.0, 147.2. ESI-MS (m/z): calcd. for $C_{12}H_{10}BNO_4$: 242.1 [M-H]$^+$; Found: 242.1.

Compound 5: To the mixture of compound **6** (146 mg, 0.6 mmol), 6-bromoquinolin-2(1*H*)-one (112 mg, 0.5 mmol) and K_2CO_3 (58 mg, 1.25 mmol) in 10 mL mixed solvents of dioxane/H_2O (4/1, v/v) was added Pd(dppf)Cl_2 (58 mg, 0.05 mmol) under N_2 atmosphere, then the mixture was bubbled with N_2 for 10 min and heated at 100 ℃ for another 6 h. Monitored by TLC, when complete, the mixture was cooled to ambient temperature and poured into water (50 mL) and extracted with CH_2Cl_2 (3 × 50 mL). The combined organic layers were sequentially washed with water and saturated NaCl aqueous solution (50 mL, each), and dried over anhydrous Na_2SO_4, and the solvent was removed by reduced pressure. The residue was subjected to column chromatography (eluent: CH_2Cl_2/CH_3OH 50/1, v/v) to give desired yellow solid in the yield of 25%. ^{1}H NMR (400 MHz, DMSO-d_6): δ 6.56 (d, J = 9.6 Hz, 1H), 7.42 (d, J = 9.6 Hz, 1H), 7.88-7.94 (m, overlapping, 5H), 7.99 (d, J = 9.6 Hz, 1H), 8.04 (d, J = 8.4 Hz, 2H), 8.10 s, 1H), 8.33 (d, J = 8.4 Hz, 2H), 11.84 (s, 1H). ^{13}C NMR (100 MHz, DMSO-d_6): δ 116.3, 120.0, 122.8, 124.6, 126.2, 127.6, 128.1, 128.3, 129.4, 133.1, 136.9, 139.0, 140.3, 140.8, 146.5, 147.1, 162.4. HR-MS (MALDI-TOF, m/z): calcd. for $C_{21}H_{15}N_2O_3$: 343.1083 [M+H]$^+$; Found: 343.1080.

Compound 4: To the solution of compound **5** (38 mg, 0.1 mmol) in 8 mL DMF was added Pd/C (10 wt％, 18 mg), then the mixture was stirred under H_2 atmosphere (1.0 atm) at ambient temperature overnight. Monitored by TLC, when complete, the precipitate was filtered and washed thoroughly with DMF (20 mL) until the filtration was almost no color. Then the combined organic layer was concentrated under reduced pressure and the crude product was recrystallized from DMSO/CH_3OH to give desired product in the yield of 50％. ^1H NMR (400 MHz, DMSO-d_6): δ 5.26 (s, 2H), 6.55 (d, J = 9.2 Hz, 1H), 6.67 (d, J = 8.4 Hz, 2H), 7.38–7.44 (m, overlapping, 3H), 7.65 (d, J = 8.4 Hz, 2H), 7.72 (d, J = 8.4 Hz, 2H), 7.88 (d, J = 8.8 Hz, 1H), 8.00 (t, J = 9.6 Hz, 2H), 11.82 (s, 1H). ^{13}C NMR (100 MHz, DMSO-d_6): δ 114.7, 116.2, 120.0, 122.7, 125.6, 126.2, 127.1, 127.5, 127.8, 129.1, 133.8, 136.8, 138.5, 139.9, 140.9, 148.9, 162.3. HR-MS (MALDI-TOF, m/z): calcd. for $C_{21}H_{17}N_2O$: 313.1341 $[M+H]^+$; Found: 313.1340.

Synthesis of [1,1':4',1":4",1"':4"',1"":4"",1""'-Sexiphenyl]-4,4""'- diamine[2]

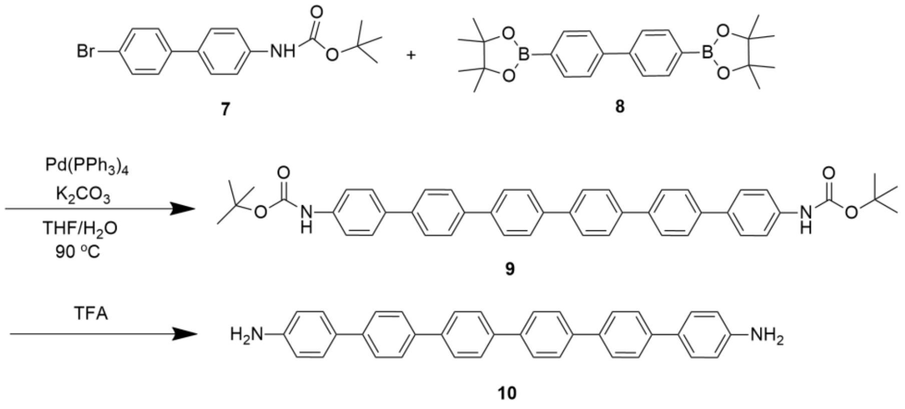

Compound 10: To a three-necked schlenk flask (150 mL) were added compound **7** (1.04 g, 3 mmol), compound **8** (0.41 g, 1 mmol), K_2CO_3 (1.80 g, 13.0 mmol), and $Pd(PPh_3)_4$ (58 mg, 0.05 mmol). The flask was evacuated and back-filled with inert argon gases over three times, after which a degassed THF/H_2O co-solvent (15 mL/7 mL) were injected into the flask through syringe. The mixture was heated up to 90 ℃ and stirred for 14 h. After cooling down to room temperature, the reaction mixture was poured into water, and then extracted with CH_2Cl_2. The organic layer was dried over anhydrous $MgSO_4$ and filtered off from an insoluble fraction. The solvent was removed under reduced pressure. Then the crude product was purified by silica gel

column chromatography (eluent, petroleum ether:ethyl acetate = 9:1) to give compound **9** as a light yellow powder. This solid was then dissolved in a mixture of CH_2Cl_2 (3 mL) and TFA (3 mL) and stirred for 2 h at room temperature. The solvent was removed under vacuum to give the target compound **10** (0.37 g, 75%) as a light yellow powder. ^{1}H NMR (400 MHz, DMSO-d_6) δ 7.99–7.60 (m, 16H), 7.47 (d, J = 7.4 Hz, 4H), 6.67 (d, J = 6.7 Hz, 4H), 5.28 (s, 4H). HR-MS (MALDI-TOF, m/z): calcd. for $C_{36}H_{28}N_2$: 488.2252 [M^+]; Found: 488.2265.

Supplementary Note 2. The Single Molecule-Connection Analyses

Referring to previously reported results[3], the number of junctions that contribute to charge transport can be figured out by calculating the probability of the connected devices with *n*-rejoined junctions (G_n) with the binomial distribution and the optimized connection yields ($Y_{connection}$):

$$G_n = \frac{m!}{n!(m-n)!} p^n (1-p)^{m-n} \qquad n = 0, 1, 2 \cdots, m \qquad (1)$$

$$Y_{connection} = 1 - G_0 = 1 - \frac{m!}{0!(m-0)!} p^0 (1-p)^m \qquad (2)$$

where m is the number of graphene point contact pairs (210 in this case), p is the connection success rate, and G_0 is the probability of devices without any connection. According to statistic results, the optimized connection yield ($Y_{connection}$), the fraction of the graphene point contact devices that showed increased conductance after EDCI coupling reaction, was 18%.

The ratio of single-junction devices to the overall reconnected devices is about 90%, indicating that, in most cases, charge transport in these devices mainly exists in a single-molecule junction.

Supplementary References

1 Cao, Y. *et al.* Building high-throughput molecular junctions using indented graphene point contacts. *Angew. Chem. Int. Ed.* **51**, 12228−12232 (2012).

2 Zang, Y. *et al.* Electronically transparent Au−N bonds for molecular junctions. *J. Am. Chem. Soc.* **139**, 14845–14848 (2017).

3 Baruah, P. K., Gonnade, R., Phalgune, U. D. & Sanjayan, G. J. Self-assembly with degenerate prototropy. *J. Org. Chem.* **70**, 6461−6467 (2005).
